# Inverted perovskite solar cells with enhanced lifetime and thermal stability enabled by a metallic tantalum disulfide buffer layer†

**DOI:** 10.1039/d1na00172h

**Published:** 2021-04-09

**Authors:** Konstantinos Chatzimanolis, Konstantinos Rogdakis, Dimitris Tsikritzis, Nikolaos Tzoganakis, Marinos Tountas, Miron Krassas, Sebastiano Bellani, Leyla Najafi, Beatriz Martín-García, Reinier Oropesa-Nuñez, Mirko Prato, Gabriele Bianca, Iva Plutnarova, Zdeněk Sofer, Francesco Bonaccorso, Emmanuel Kymakis

**Affiliations:** Department of Electrical & Computer Engineering, Hellenic Mediterranean University (HMU) Heraklion 71410 Crete Greece krogdakis@hmu.gr kymakis@hmu.gr; Institute of Emerging Technologies (i-EMERGE) of HMU Research Center Heraklion 71410 Crete Greece; BeDimensional SpA Via Lungotorrente Secca 3d 16163 Genova Italy; Graphene Labs, Istituto Italiano di Tecnologia via Morego 30 Genova 16163 Italy; CIC nanoGUNE Tolosa Hiribidea, 76 20018 Donostia-San Sebastian Spain; Department of Materials Science and Engineering, Uppsala University Box 534 751 03 Uppsala Sweden; Materials Characterization Facility, Istituto Italiano di Tecnologia via Morego 30 16163 Genova Italy; Dipartimento di Chimica e Chimica Industriale, Università degli Studi di Genova via Dodecaneso 31 16146 Genoa Italy; Department of Inorganic Chemistry, University of Chemistry and Technology Prague Technická 5 166 28 Prague 6 Czech Republic

## Abstract

Perovskite solar cells (PSCs) have proved their potential for delivering high power conversion efficiencies (PCE) alongside low fabrication cost and high versatility. The stability and the PCE of PSCs can readily be improved by implementing engineering approaches that entail the incorporation of two-dimensional (2D) materials across the device's layered configuration. In this work, two-dimensional (2D) 6R-TaS_2_ flakes were exfoliated and incorporated as a buffer layer in inverted PSCs, enhancing the device's PCE, lifetime and thermal stability. A thin buffer layer of 6R-TaS_2_ flakes was formed on top of the electron transport layer to facilitate electron extraction, thus improving the overall device performance. The optimized devices reach a PCE of 18.45%, representing a 12% improvement compared to the reference cell. The lifetime stability measurements of the devices under ISOS-L2, ISOS-D1, ISOS-D1I and ISOS-D2I protocols revealed that the TaS_2_ buffer layer retards the intrinsic, thermally activated degradation processes of the PSCs. Notably, the devices retain more than the 80% of their initial PCE over 330 h under continuous 1 Sun illumination at 65 °C.

## Abstract 2

An ultra-thin 2D TaS_2_ buffer layer on top of the electron transport layer improves the efficiency,the lifetime and the thermal stability of inverted perovskite solar cells.

## Graphical abstract

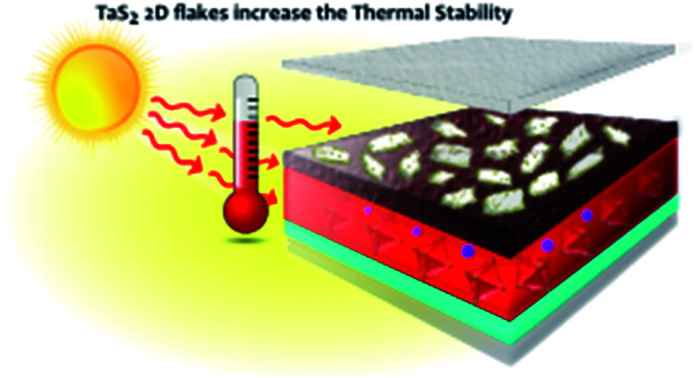

## Introduction

Organic–inorganic hybrid perovskite solar cells (PSCs) have revealed their potential as excellent solar energy conversion devices, reaching a power conversion efficiency (PCE) of 25.2%.^1^ The biggest challenge for the further deployment of PSCs is to attain enhanced lifetime stability without reducing device PCE while up-scaling active area and manufacturing processes. Engineering approaches to tackle these issues include the incorporation of two-dimensional (2D) interlayers (*e.g.*, graphene^2^ and transition metal dichalcogenides (TMDs))^3^ – a process applicable also in large-area modules^4–6^ – the optimization of the doping and surface functionalization of 2D interlayers,^7^ as well the possibility of integrating passivation layers^8^ such as 2D insulators.^9^ Therefore, the flexible design of printable PSCs with integrated 2D materials offers many possibilities to discover new properties and functionalities, and constitutes a perfect match for providing power in autonomous Internet of Things (IoT) and wearable systems.^10^

In particular, the engineering of PSC's charge transport layers (CTLs) has a pivotal role in achieving high PCEs and extended device lifetimes.^11,12^ Thanks to the plethora of their properties, 2D materials are excellent candidates for tuning and/or replacing the hole transport layer (HTL) or electron transport layer (ETL) of PSCs.^13–15^ For instance, graphene has been extensively used in PSCs as dopant for CTLs for enhancing their carriers' mobility.^16–19^ Meanwhile, 2D TMDs have been recently incorporated in PSCs due to their unique electrical and optical properties, allowing the energey levels to be aligned across cell's layers.^16,20^ Among 2D materials, group-VI TMDs (*e.g.*, WSe_2_, MoS_2_, MoSe_2_) and group-XIV metal chalcogenides (*e.g.*, SnS_2_) have been integrated in PSCs using various approaches. Specifically, 2D MoS_2_ flakes were used as interlayer over the HTL in inverted^21–24^ and normal PSCs,^7,25^ increasing the PCE and the stability, while approaches for HTL replacement were also demonstrated.^26^ Few-layer flakes of SnS_2_ were used as ETL material in planar PSC structures, exhibiting PCE values up to 20.1%,^27^ while WSe_2_ interlayer over PEDOT:PSS was reported to increase the PCE from 13.8% to 16.2%.^28^ Finally, inverted PSCs were fabricated through the employment of MoSe_2_ as HTL^29,30^ or as buffer layer.^31^

Differently from the most investigated TMDs, group-V TMDs (*e.g.*, TaS_2_, NbS_2_ and VS_2_) can be found in metal-like phases^32,33^ such as 2H-, 3R- and 6R polytypes for Nb- or Ta-based TMDs,^34,35^ and 1T-polytypes for V-based TMDs.^36,37^ Nevertheless, their unique properties have been rarely used in photovoltaic (PV) systems, and only few studies have recently reported the successful integration of group-V TMDs (mainly TaS_2_ and NbS_2_) in PSCs.^38,39^ These investigations mainly aimed to replace traditional CTLs by bulk or 2D group-V TMDs. For instance, a thin film of metallic TaS_2_ has been recently used as back contact in Cu_2_BaSnS_4_ solar cells as alternative to Mo/MoS_2_.^40^ The potential of incorporating 2D TaS_2_ in PVs was demonstrated by inserting TaS_2_ nanosheets as HTL (oxidized flakes) or ETL in normal and inverted organic solar cells, respectively, leading to a PCE enhancement – especially for the case of inverted configurations. The PCE improvement was attributed to the favourable energy level alignment between the active layer and the TaS_2_ CTLs.^41^ Following a similar approach, TaS_2_ nanosheets were used to replace SnO_2_ that served as ETL in normal PSCs, leading to a PCE as high as 15.23%.^38^

In this study, differently from previous works, we investigated the incorporation of 2D TaS_2_ flakes in inverted PSCs as efficient buffer layer on top of the ETL, improving both the PCE and the stability of reference devices. More in detail, TaS_2_ flakes were produced through an up-scalable ultrasonication-assisted liquid-phase exfoliation (LPE) process of the corresponding bulk 6R-TaS_2_ crystals and then were deposited on the ETL, *i.e.*, PC_70_BM. The buffer layer formed by TaS_2_ flakes was optimized by varying the number of consequent spin coatings (SCs). The PSCs incorporating the TaS_2_ buffer layer demonstrated a PCE value of 18.45%, corresponding to a 12% improvement compared to the reference device (PCE = 17.66%). The enhanced performance is attributed to the excellent charge transport properties and suitable work function (*W*_F_) of TaS_2_ flakes, matching the energy level of the in-contact materials. More importantly, the TaS_2_-enabled devices exhibited an improved lifetime and thermal stability when tested under ISOS-L2, ISOS-D1I and ISOS-D2I protocols. The optimized devices showed a degradation rate of 0.061% PCE per h and retained more than the 80% of their initial PCE for 330 h under continuous 1 Sun AM1.5G illumination.

## Experimental

### Synthesis and exfoliation of 6R-TaS_2_ crystals

6R-TaS_2_ crystals were prepared through direct synthesis from elements, *i.e.*, Ta and S, following protocols previously reported in literature.^42^ Experimentally, amounts of Ta (99.999%, <6 μm) and S (99.9%, <100 μm) powders (Strem Chemicals, Inc.), with a S : Ta stoichiometry of 2 : 1, were placed in a quartz glass ampoule (20 mm × 120 mm). Once evacuated until a pressure of 1 × 10^−3^ Pa, the ampoule was sealed using an oxygen–hydrogen welding torch. The ampoule was heated to 450 °C for 12 h, and subsequently heated to 600 °C for 48 h, and up to 900 °C for 48 h. Afterwards, the ampoule was cooled down to room temperature over 24 h. The heating rate was +5 °C min^−1^ for all the heating steps. The TaS_2_ flakes were produced by ultrasonication-assisted LPE of fragments of 6R-TaS_2_ crystals,^42–44^ followed by sedimentation-based separation (SBS),^45^ in anhydrous isopropanol (IPA). Briefly, 50 mg of crystal fragments were inserted in 50 mL of anhydrous IPA. The resulting mixture was ultrasonicated in a sonicator (Branson® 5800 cleaner, Branson Ultrasonics) for 6 h. Then, the dispersion was ultracentrifuged using a Beckman Coulter centrifuge (Optima™ XE-90 with a SW32Ti rotor) at 2700*g* for 20 min at 15 °C to separate the exfoliated materials (as supernatant) from the unexfoliated bulk crystals (sediment). Lastly, the TaS_2_ flakes were collected by pipetting 80% of the supernatant. The concentration of the so-produced TaS_2_ nanoflakes dispersion was fixed to 1 mg mL^−1^ by adjusting the amount of IPA.

### Materials characterization

Scanning electron microscopy (SEM) measurements of the as-synthetized 6R-TaS_2_ crystals were acquired using JEOL® JSM-6490LA SEM equipped with an energy-dispersive X-ray spectroscopy (EDS) detector, operating at 20 kV. The samples were imaged without any metal coating or pre-treatment.

Bright-filed transmission electron microscopy (BF-TEM) measurements of the TaS_2_ flakes were performed with a JEM 1011 (JEOL) TEM (thermionic W filament), operating at 100 kV. ImageJ software (NIH) and OriginPro 9.1 software (OriginLab) were used to analyse the images and to perform the statistical analysis of the flakes' lateral dimension data, respectively. The samples were fabricated by depositing the LPE-produced TaS_2_ flake dispersions onto ultrathin C-on-holey C-coated Cu grids. The samples were rinsed with deionized water and subsequently dried overnight under vacuum before to their imaging.

Atomic force microscopy (AFM) measurements were performed using a Nanowizard III (JPK Instruments, Germany), mounted on an Axio Observer D1 (Carl Zeiss, Germany) inverted optical microscope. The images were acquired using a PPP-NCHR cantilevers (Nanosensors, USA), which have a tip with a nominal diameter of 10 nm. The image acquisition was performed using a drive frequency of ∼295 kHz. Intermittent contact mode was used to record the image over an area of 2.5 × 2.5 μm^2^ (512 × 512 data points), using a scan rate of 0.7 Hz. The working set point was set above 70% of the free oscillation amplitude. JPK Data Processing software (JPK Instruments, Germany) and OriginPro 9.1 software were used to elaborate the height profiles and to perform the statistical analysis of the flakes' thickness data. The samples were produced by depositing the LPE-produced TaS_2_ flake dispersions on substrates of mica (G250-1, Agar Scientific Ltd.). Before the measurements, the samples were dried under vacuum overnight.

X-ray diffraction (XRD) characterization was carried out using a PANalytical Empyrean with Cu Kα radiation. The samples were produced by depositing powder of 6R-TaS_2_ crystals or LPE-produced TaS_2_ flake dispersions onto Si/SiO_2_ substrates. Before the measurements, the samples were dried under vacuum overnight to remove moisture or solvent residuals.

Raman spectroscopy measurements were carried out using a Renishaw microRaman Invia 1000 mounting a 50× objective. The excitation wavelength the incident power on the samples were 532 nm and 1 mW, respectively. The samples were prepared following the procedure described for the XRD characterization.

Kelvin probe force microscopy (KPFM) images were acquired in air with a XE7 AFM (Park System, Korea) and low-noise lock-in amplifier SR830 DSP (Stanford research systems, USA) operating in AM mode equipped with Au-coated PPP-NCSTAu cantilevers (Nanosensors, Switzerland) having a tip diameter less than 50 nm. The topography images were collected in non-contact mode using the resonant oscillation of the cantilever (160 kHz) and the potential images were collected using an AC modulation voltage of 0.5 V at 17 kHz applied to the tip. The scan rate for the acquisition of the images was 0.1–0.2 Hz. The samples were prepared by spin-coating a droplet of the TaS_2_ flakes dispersion onto ITO.

Ultraviolet photoelectron spectroscopy (UPS) with He I (*hν* = 21.2 eV) radiation was performed using a Kratos Axis Ultra^DLD^ spectrometer, operating at <10^−8^ mbar. A −9.0 V bias was applied to the sample to precisely determine the low kinetic energy cut-off.

### Device fabrication

Pre-patterned ITO/glass substrates were cleaned in ultrasonic bath using deionised water, acetone, and IPA. Then, the samples were transferred in N_2_ glovebox, where they underwent a UV–ozone treatment for 15 min. Thin (∼10 nm) films of poly[bis(4-phenyl)(2,4,6-trimethylphenyl)amine] (PTAA) (Solaris *M*_w_ = 20–70 kDa) were prepared by spin coating a 2 mg mL^−1^ PTAA solution in toluene at an angular speed of 6000 rpm. The PTAA films were annealed at 110 °C for 10 min. The perovskite solution was prepared by mixing 0.2 M methylammonium bromide (MABr) (GreatCell Solar), 1.14 M formamidinium iodide (FAI) (GreatCell Solar), 0.2 M PbBr_2_ (TCI America) and 1.24 M PbI_2_ (TCI America) in 4 : 1 v/v anhydrous dimethylformamide (DMF) (99.8%, Sigma Aldrich) : dimethyl sulfoxide (DMSO) (99.9%, Sigma Aldrich) and then adding 5 vol% from a 1.5 M CsI stock solution and 4 vol% from 1.5 M RbI stock solution. The perovskite layers were spin coated on the PTAA film at 6000 rpm for 45 s. 10 s prior to the end of the spinning process, 200 mL of anhydrous chlorobenzene (CB) (99.8%, Sigma Aldrich) was dropped onto the spinning perovskite film. Subsequently, the samples were immediately annealed for 45 min on a hotplate preheated at 100 °C. Next, thin-layers of PC_70_BM (99%, Solenne) were deposited by spin coating a 20 mg mL^−1^ PC_70_BM solution in anhydrous CB onto the perovskite at 1000 rpm. The PSCs were then completed following a procedure described in ref. 8. Briefly, the TaS_2_ flakes were deposited from their dispersions onto PC_70_BM through consecutive SCs (up to five). Afterwards, 45 mL of 0.5 mg mL^−1^ bathocuproine (BCP) (96%, Sigma Aldrich) prepared in IPA (99.5% extra dry, ACROS Organics) was spin coated onto the TaS_2_ buffer layers. Finally, a 100 nm-thick Ag top electrode was deposited by vacuum thermal evaporation.

### Device characterization

The PSCs were evaluated under an inert atmosphere using an ABB solar simulator (Sol1A, Oriel) equipped with a 450 W Xe lamp and an AM1.5G filter. The intensity was calibrated at 100 mW cm^−2^ using a KG3-window Si reference cell. The *J*–*V* curves were recorded at a constant scan rate of 20 mV s^−1^ using a multiplexor test board system (Ossila), and no device preconditioning was applied before the measurements. A black metallic aperture mask was used during each measurement to set the active area of the fabricated devices at 0.04 cm^2^ and to reduce the influence of the scattered light. The external quantum efficiency (EQE) spectra were recorded using a QE-T system from Enlitech. A chopping frequency of 60 Hz was used. The calibration of the light intensity was performed using a quartz-window Si solar cell. The integrated current density was calculated by integrating the product between the spectral response of the test cell and the reference AM1.5G solar spectrum. Optoelectrical characterization was performed with a transient module of ARKEO measurement platform (Cicci Research s.r.l.). Transient photovoltage (TPV) experiments were performed in small perturbation mode by confining the intensity of the light pulse to less than 10% of the background voltage, unaltering the equilibrium of the field induced by the background bias. The voltage decay of the measured devices was fitted by an exponential decay with a time constant that directly reflects the lifetime of the charge carriers. Transient photocurrent (TPC) experiments were performed in both large and small perturbation regimes. Large perturbations were induced over 200 ms under a 0.8 duty cycle, while small perturbations were loaded with a 0.001 cycle and passed through an external circuit of 50 ohm resistance. Both the signals of open circuit voltage, *V*_oc_, (for TPV) and short circuit current, *J*_sc_, (for TPC) were monitored after passing them through voltage and impedance amplifiers. To observe the photoinduced charge extraction through linearly increasing voltage (photo-CELIV) measurements, a 470 nm fast LED source driven by a 100 mA current and exhibiting a Lambertian radiation pattern was used. The relaxation pulse width was set to 20 ms, charged by a 50 000 V s^−1^ ramp, following a 13 ms delay after the injection pulse. The collected signals were processed through a transimpedance amplifier and passed through a 100 MHz bandwidth digitizer running in single-shot mode. The lifetime behaviour of the PSCs was monitored using an ISOS testing laboratory from InfinityPV in ISOS-L2 operation mode. The devices were first encapsulated with a piece of glass and a UV-curable epoxy as an adhesive (Ossila E132). Then, the devices were placed inside the test chamber and exposed to continuous illumination. The apparatus was equipped with a solar simulator using a metal halide light source simulating the AM1.5 G spectrum in the range of 300–900 nm. The light intensity was calibrated at 100 mW cm^−2^ using a Si reference cell. The humidity in the test chamber was below 15%, while the temperature was over 65 °C. Between the *J*–*V* measurements, the devices were left in open circuit condition. The shelf lifetime measurement of the unencapsulated devices was performed in the glove box either at room temperature (ISOS D1I) or at 65 °C (ISOS D2I).

## Results and discussion

The as-synthesized 6R-TaS_2_ crystal was characterized by SEM-EDS measurements (see ESI, Fig. S1a–c†), showing crystal fragment with nearly straight borders and a S : Ta atomic ratio of 1.9 ± 0.2, which is similar to the values previously measured for TaS_2_ produced with similar protocols.^42,44,46,47^ Fig. S1d† shows a SEM image zooming a crystal edge, evidencing the layered structure the 6R-TaS_2_ crystals. Transmission electron microscopy and AFM measurements were performed to evaluate the lateral size and thickness of the TaS_2_ flakes produced through LPE of fragments of 6R-TaS_2_ crystals in anhydrous IPA. Fig. S2a† shows a BF-TEM image of representative TaS_2_ flakes, exhibiting irregular shapes with flat surfaces and nearly straight edges. The data of the lateral size of the flakes follow a log-normal distribution peaked at ∼25 nm, with an average value of 87.6 nm (Fig. S2b†). Fig. S2c† reports an AFM image of representative TaS_2_ flakes. The statistical analysis of the thickness data indicate that they follow a log-normal distribution peaked at ∼1.7 nm (Fig. S2d†). Since the AFM thickness of TaS_2_ monolayer typically lies between 0.4 and 0.9 nm (depending on the TaS_2_/substrate interaction and AFM instrumentation),^48,49^ these results indicate that the exfoliated TaS_2_ sample mainly consists of single-/few-layer flakes. The structural properties of the TaS_2_ flakes were evaluated through XRD and Raman spectroscopy measurements. Fig. S3a† reports the XRD pattern of the TaS_2_ flakes in comparison to the one recorded for the native bulk crystal. Both the XRD patterns shows reflections which match those of the 6R phase ((ICSD-52117), although a secondary 2H phase (ICSD-68488) coexists with a marginal contribution, as observed by previous studies on 6R-TaS_2_ polytypes.^42^ Fig. S3b† shows the Raman spectra of both bulk and exfoliated TaS_2_ samples, further confirming their crystal structure. Noteworthy, 6R-TaS_2_ polytype exhibits Raman modes similar to those of 2H polytype, namely the out-of-plane vibration A_1g_ mode at ∼380 cm^−1^, the in-plane vibrational E^1^_2g_ mode at ∼280 cm^−1^ and the broad second-order peak attributed to two-phonon process at ∼180 cm^−1^.^50,51^ The TaS_2_ flakes retain the position of the A_1g_ mode of the bulk crystals. Differently, E^1^_2g_ is blue-shifted by ∼17 cm^−1^, because of the decrease of long-range Coulomb interlayer interactions with decreasing the number of layers.^52^ Moreover, the two-phonon peak is red-shifted by 22 cm^−1^, which may be consequence of the emergence of the E_1g_ mode.^53^ The latter can be activated by the symmetry breaking caused by interaction of single/few layer nanoflakes with SiO_2_/Si substrate.^54^ The layers with octahedral Ta coordination may also contribute with Raman mode resembling those exhibited by 1T-TaS_2_ polytypes, which typically shows peaks at ∼250, ∼310 and ∼380 cm^−1^.^55–58^

To investigate the energetics of the TaS_2_ flakes, the latter were deposited on ITO and characterized by KPFM. Fig. S4† displays a 3D representation of surface topography including an overlayer of the Contact Potential Difference (CPD). Interestingly, at the places where TaS_2_ flakes were deposited, the CPD increase by 15 mV compared to the substrate values. These data indicate that the *W*_F_ of the TaS_2_ flakes is lower than the one of the ITO.^59^ Lastly, UPS measurements were performed to quantitatively estimate the *W*_F_ of a film of TaS_2_ flakes. Fig. S5a† shows the secondary electron cut-off region of the spectrum indicating a cut-off energy of ∼17.1 eV, corresponding to a *W*_F_ of 4.1 eV. Noteworthy, the LPE process is effective to reduce the *W*_F_ of the 6R-TaS_2_ bulk crystal, which is 3.9 eV (Fig. S5b†). This means that the exfoliation of the material can tune the energy barrier between the PC_70_BM LUMO (4.0 eV) to improve the electron extraction process from PC_70_BM to 6R-TaS_2_ (see the details on the PSC structure in the next section). Although the LPE process duration may further improve the electron extracting properties of the 6R-TaS_2_ flakes, the concomitant reduction of the lateral dimension of the flakes may negatively affect the protective properties of the 6R-TaS_2_ flakes against volatile species and ion migration, as demonstrated later in the text. Therefore, we limited the exfoliation of the flakes without prolonging further the LPE process. Fig. S5c and d,† show the regions near the Fermi energy level of the UPS spectra for both bulk and exfoliated 6R-TaS_2_ crystals. The presence of an “edge” centred at the zero of the binding energy scale confirms the metallic character of the 6R phase of TaS_2_, in agreement with previous literature.^42^

### Structural and morphological characterization of PSCs

**Fig. 1:**
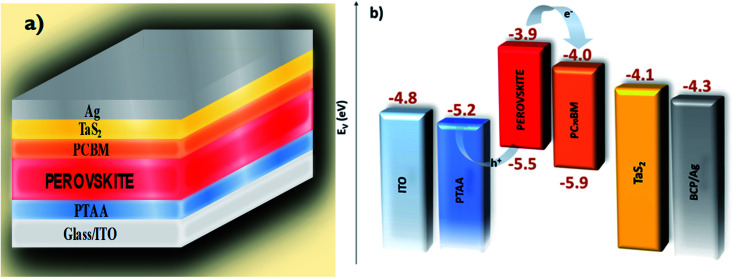
(a) Schematic illustration of an inverted PSC incorporating 6R-TaS_2_ flakes, with the following layered stack: Glass/ITO/PTAA/Perovskite/PC_70_BM/TaS_2_/Ag. (b) Energy level diagram of the material stack in the PSC.

Fig. 1 shows a schematic illustration of an inverted PSC, which consists of a glass/ITO/PTAA/perovskite/PC_70_BM/TaS_2_/BCP/Ag material stack. Based on the characterization of the TaS_2_ flakes (Fig. S1–S5†), the *W*_F_ of 4.1 eV and the metallic character of the material indicate that TaS_2_ buffer layers over the PC_70_BM can effectively passivate interfacial defects and pin-holes,^60–62^ while improving the energy level alignment at the interface between the ETL and Ag electrode^63^ that has a *W*_F_ of ∼4.3 eV.^64^ Hereafter, PSC-1, -2, -3 and -5 refers to a device incorporating TaS_2_ buffer layers obtained through 1, 2, 3 and 5 SCs, respectively.

**Fig. 2:**
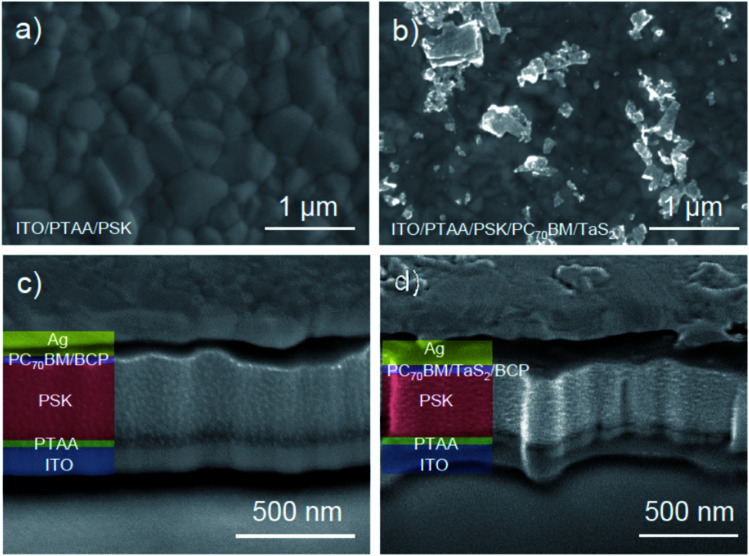
Representative top-view and cross-sectional SEM images of PSC-Ref (panels a, c) and PSC-2 (panels b, d). (a) Top-view of perovskite surface prior to PCBM deposition in PSC-Ref. (b) Top-view of the 6R-TaS_2_ buffer layer on top of the PC_70_BM layer. (c) Cross-sectional SEM image of PSC-Ref. (d) Cross-section SEM image of PSC-2 incorporating the TaS_2_ buffer layer. False colouring was used for the different layers of the structure: ITO/PTAA/perovskite (PSK)/PC_70_BM/Ag.

Scanning electron measurements (FEI Helios Nanolab 450S microscope −5 kV and 0.2 nA imaging conditions) were performed to elucidate the layered structures of both reference PSC without TaS_2_ buffer layer (PSC-Ref) and PSC-2 (Fig. 2a, c and b, d, respectively). Fig. 2a shows the surface of the perovskite layered in PSC-Ref, revealing crystal grain sizes of *ca.* 250 nm. Fig. 2b shows the top-view of the TaS_2_ buffer layer deposited over PC_70_BM layer. Since most of the flakes have nanometric thicknesses (see Fig. S2c and d†), only occasional thick TaS_2_ flakes are clearly distinguished by SEM imaging. Nevertheless, SEM-EDS analysis (FEI Quanta 250 FEG microscope, acquiring at 30 kV) (Fig. S6b and c†) confirmed the homogeneous presence of TaS_2_ flakes on the PC_70_BM surface. Fig. 2c depicts a cross-section of the layered structure (made by focused ion beam, dual-beam FEI Helios Nanolab 450S microscope) of the complete reference device, showing a perovskite layer with a thickness of about 350–400 nm. As shown in the cross-sectional SEM image of PSC-2 (Fig. 2d), the device structure is not significantly affected by the incorporation of the few-nm thick TaS_2_ buffer layer.

### Steady state photovoltaic characterization of PSC devices

**Fig. 3:**
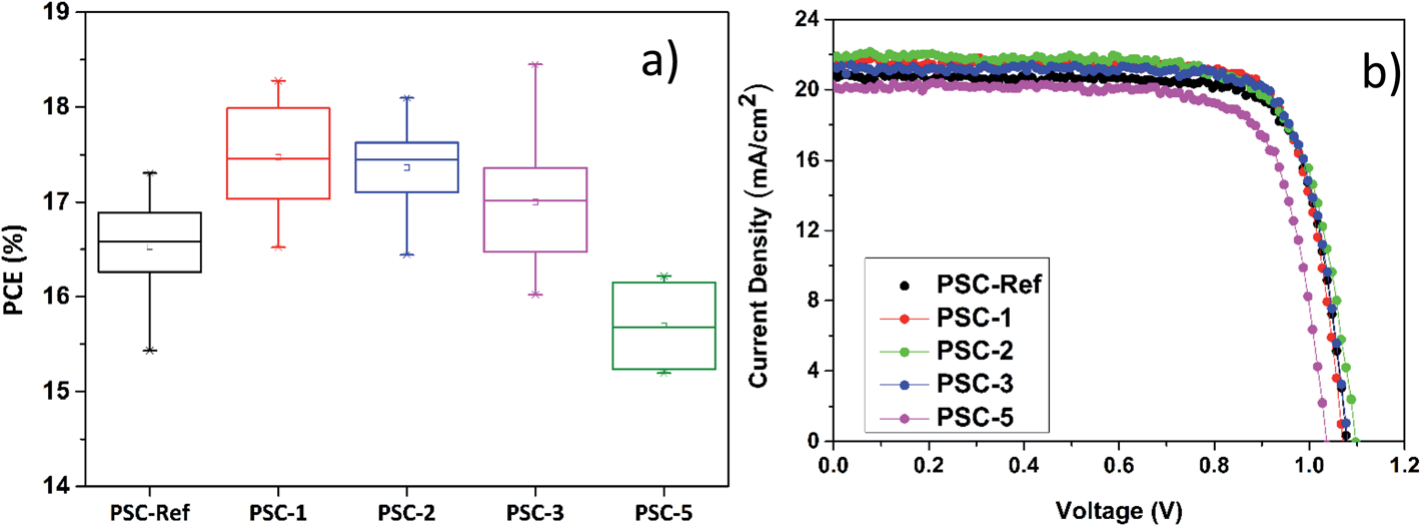
Photovoltaic performance of the reference PSC (PSC-Ref) and the PSCs incorporating TaS_2_ flakes as a buffer layer (PSC-*X*). (a) Box chart of PCE performance for PSC-Ref, PSC-1, PSC-2 PSC-3 and PSC-5, and (b) the *J*–*V* curves measured for the champion devices for each PSC configuration.

The PV performance of the investigated PSCs was evaluated as a function of the number of consecutive SCs of the TaS_2_ flakes dispersion on top of the PC_70_BM. As shown in Fig. 3a, the PV performance of the devices is improved upon TaS_2_ flakes deposition, except when the total number of SCs is more than three. The maximum PCE performance was recorded for PSC-1 and PSC-2 devices (average PCE of 17.5% and 17.4%, respectively), while the average PCE of PSC-3 (17.3%) also remained higher than the one recorded for PSC-Ref (∼16.6%). By increasing the number of the SCs to more than 3, the average PCE decreases to 15.74% because of the enhanced electron scattering rate as the thickness of the TaS_2_ buffer layer increases. Moreover, the excessive IPA exposure of the device for 5 SCs is detrimental for the device performance.^31^Fig. 3b shows the *J*–*V* curves of the champion PSC-1, PSC-2, PSC-3, PSC-5 and PSC-Ref, while Table 1 summarizes the main device PV parameters extracted from the *J*–*V* curves analysis, *i.e.*: average and maximum values of PCE, open circuit voltage (*V*_oc_), short circuit current density (*J*_sc_) and fill factor (FF). The average PCE improves from 16.57% in PSC-Ref to 17.53%, 17.36% and 17.30% for the PSC-1, PSC-2 and PSC-3, respectively and drops to 15.74% in PSC-5. The champion device (PSC-3) achieved a PCE up to 18.45%, representing a +12% PCE improvement compared to the best PSC-Ref (PCE = 17.66%). All devices exhibited negligible hysteresis, as revealed by their reverse *J*–*V* scan analysis shown in Fig. S7 and Table S1.† Moreover, the steady-state PCE of the devices was quite stable, as shown in Fig. S7b,† where the *J*_sc_ stabilized after 120 s after a slight initial increase. Fig. S7c† shows the EQE spectrum of the most performant device together with the calculated current density, which matches the *J*_sc_. Fig. S8† shows the photoluminescence (PL) measurements of samples fabricated up to perovskite layer, as well as samples containing also PC_70_BM and TaS_2_ layers. The addition of PC_70_BM ETL on top of the perovskite causes PL quenching because of a more efficient electron extraction by the PC_70_BM. Meanwhile, the PL peak is red-shifted from 790 nm in the ETL-free sample to 793 nm. The red-shift can be attributed to an enhanced photon recycling due to the metallic 2D-flakes.^65,66^ The incorporation of the TaS_2_ buffer layer does not increase further the PL quenching.

**Table 1:** Photovoltaic parameters of the investigated PSCs extracted from their corresponding *J*–*V* curves. The errors are calculated from device statistics. The values in the brackets correspond to the champion devices

	PCE (%)	*V*_oc_ (V)	*J*_sc_ (mA cm^−1^)	FF (%)
PSC-Ref	16.57 ± 0.43 (17.66)	1.067 ± 0.022 (1.081)	20.71 ± 0.58 (20.84)	74.16 ± 1.66 (78.37)
PSC-1	17.53 ± 0.50 (18.28)	1.077 ± 0.021 (1.070)	21.25 ± 0.43 (21.56)	75.48 ± 2.45 (79.22)
PSC-2	17.36 ± 0.12 (18.10)	1.088 ± 0.020 (1.096)	21.41 ± 0.52 (21.98)	73.87 ± 1.87 (75.09)
PSC-3	17.30 ± 0.60 (18.45)	1.068 ± 0.020 (1.080)	21.29 ± 0.45 (21.45)	75.07 ± 2.20 (79.65)
PSC-5	15.74 ± 0.50 (16.06)	1.048 ± 0.018 (1.036)	20.01 ± 0.42 (20.15)	74.29 ± 1.58 (76.86)

### Transient photovoltage characterization of PSC devices

**Fig. 4:**
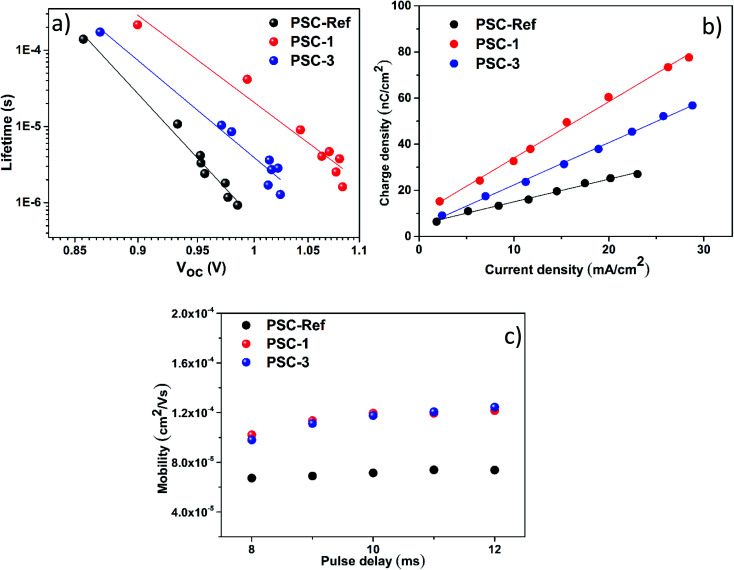
Estimation of the carrier's lifetime, the extracted charge density, as well as the effective carrier mobility, based on transient measurements. (a) Extracted carrier's lifetime from TPV decay measurements at different bias conditions. (b) Charge density extracted from TPC measurements. The lines in panels (a, b) represent linear fittings. (c) Drift mobility estimation from the photo-CELIV technique.

Transient photovoltage and TPC measurements were performed to explain the physical mechanism behind the performance enhancement reported when the TaS_2_ flakes are incorporated into the PSCs as a buffer layer. Fig. 4a shows the comparison between the lifetime decay of PSC-1, PSC-3, and PSC-Ref. The carrier lifetimes were extracted from corresponding transient tail of TPV decay curves (Fig. S9†) that follow a single exponential trend.^27^ In particular, TPV results show that the TaS_2_ buffer layer reduces the charge recombination rate and increases the charge carrier lifetime (Fig. 4a), which is consistent with the highest *V*_oc_ measured for TaS_2_-based devices (see Fig. 3a and Table 1). Furthermore, the addition of the TaS_2_ buffer layer does not significantly affect the distribution of shallow defect states (see inset panels in Fig. S9†). Transient photocurrent experiments in charge extraction mode demonstrated that the TaS_2_ buffer layer increases the overall extracted charge density, especially in the PSC-1 device (Fig. 4b). These data suggest that the metallic character of the TaS_2_ flakes (see Fig. S5b†) play a beneficial role in the charge extraction process. The measurements performed in the high perturbation regime (0.8 duty cycle) show the absence of deep trap states in all the tested samples (Fig. S10†).

The drift mobility of electrons/holes was probed using charge extraction by linearly increasing voltage (photo-CELIV) under various light pulse delay points. More in detail, the drift mobility was derived from the extracted charge represented by the part of the transient superimposed over the displacement current level (Fig. S11†). The mobility (*μ*) can be derived according to the equation:^27^
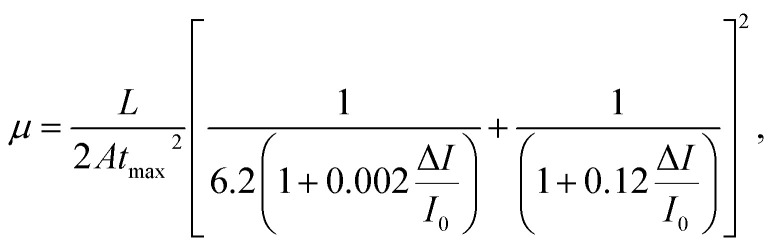
where *L* is active layer thickness, *A* is the ramp of the extraction voltage, *t*_max_ is the point of transient measurement where current reaches its peak and Δ*I*/*I*_0_ ratio corresponds to the level of charge accumulation. As shown by Fig. 4c, both PSC-1 and PSC-3 improved the charge carrier mobility by approximately half an order of magnitude compared to PSC-Ref. This effect leads to an efficient charge transfer, which agrees with the TPC analysis. Notably, the delay of the photogeneration pulses does not affect the drift mobility, which means that the charge carrier transport is optimally balanced.

### Lifetime and thermal stability measurements of perovskite devices

Various approaches have been proposed to enhance the stability of PSCs and related modules, which still suffer of significant degradation over timescale required for commercial applications.^67–69^ Here, the lifetime and shelf stability of the devices incorporating the TaS_2_ buffer layer were probed either under inert or ambient atmosphere in accordance with the updated ISOS protocols (ISOS-L2, D1I, -D2I, -D1).^70^ The updated ISOS protocols were designed to cover emerging PV technologies with distinctive characteristics (*e.g.*, light soaking effect and volatile nature of organic species) compared to commercially available solar cells. These technologies include the PSCs, which require specific treatment. Apart from measurements under continuous illumination at elevated temperature (ISOS-L2), measurements under N_2_ atmosphere were also implemented by investigating the shelf life of the devices and the thermal stability at 65 °C though ISOS-D1I and ISOS-D2l protocols, respectively. Finally, we evaluated the shelf life of our devices (unencapsulated) in ambient conditions (ISOS-D1).

**Fig. 5:**
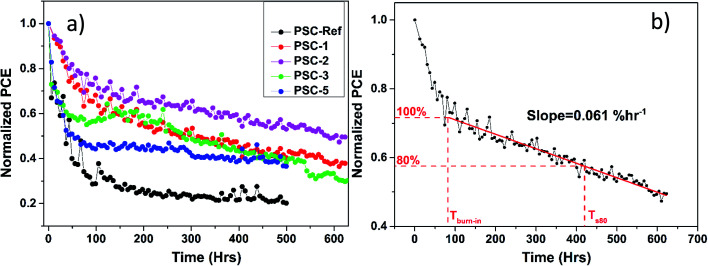
Lifetime measurements of encapsulated perovskite devices under continuous 1 sun illumination, 65 °C and 10–15% RH. (a) Long term ISOS-L2 lifetime measurements of PSC-Ref (black), PSC-1 (red), PSC-2 (magenta), PSC-3 (green) and PSC-5 (blue). (b) Normalized PCE over time for PSC-2. The degradation rate is calculated from a linear fit of the experimental data in the stabilized region. *T*_burn-in_ marks the end of the burn-in phase and *T*_s80_ denotes the time at which the PCE drops to 80% of the initial PCE at *T*_burn-in_ in the stabilized region.

Under continuous illumination at elevated temperature, the PSC-Ref exhibited a drop of its initial PCE by 50% after 35 h (*T*_50_) and lost more than the 70% of its initial PCE after 100 h (Fig. 5). The incorporation of TaS_2_ buffer layer significantly improved the device stability. In particular, the PSC-1 reported an excellent stability performance, showing a *T*_50_ of over 300 h, while its PCE did not drop below 40% even after 600 h. Notably, the best performing device PSC-2 displayed a *T*_50_ of more than 600 h. The best stability of PSC-2 can be associated to the optimal surface coverage of the TaS_2_ buffer layer obtained through two SCs. By further increasing the number of SCs, the stability of the device decreases probably because of excessive exposure of the perovskite to IPA.^27^ Nevertheless, PSC-3 and PSC-5 are still outperforming the PSC-Ref in terms of lifetime stability. These results indicate that the TaS_2_ buffer layer between the PC_70_BM and the Ag metal can serve as a protective layer, retarding device degradation under continuous illumination and thermal stresses.

As shown in Fig. 5b, the lifetime stability measurement of the most stable PSC-2 reveals a burn-in phase lasting for about 80 h. During this phase, the initial PCE drops rapidly by about 20%, and afterwards stabilizes with a slow linear decline. The burn-in behaviour is common in organic PVs^71,72^ and is even often observed in PSCs.^73–75^ However, in the latter case, the initial losses are often recovered when devices are left to rest under dark conditions.^76^ Therefore, the linear decay regime of the device was used to estimate the device lifetime. By considering the time at the end of the burn-in phase (*T*_burn-in_), the time at which the PCE drops by 20% compared to *T*_burn-in_, *T*_s80_ represents a usual metric for the PSC stability. The linear fit of the experimental data in the linear regime enables the degradation rate to be estimated. In particular, the estimated degradation slope of PSC-2 was 0.061% h^−1^. Hence, the device incorporating the TaS_2_ buffer layer are expected to retain 80% of their initial PCE for 330 h under continuous 1 Sun illumination.

**Fig. 6:**
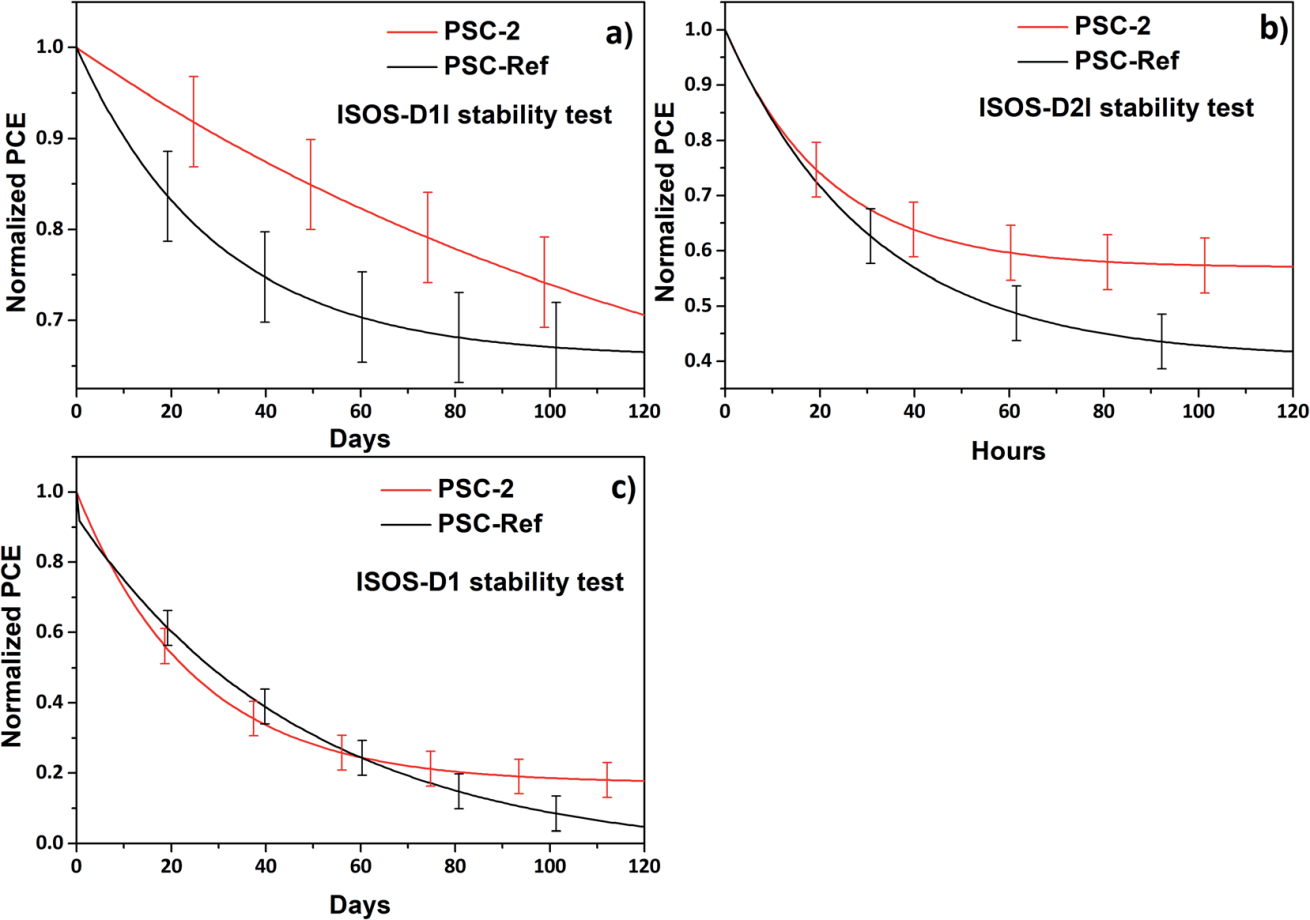
Lifetime measurements for PSC-2 (red), compared to those of PSC-Ref (black) using the following stress factors: (a) under inert atmosphere and 0% RH in the dark, (b) thermal stress at 65 °C under inert conditions and 0% RH and (c) under ambient conditions in the dark of unencapsulated cells.

Apart from ISOS-L2 protocols, the device stability was also evaluated by stressing the devices in inert atmosphere under ISOS-D1I and ISOS-D2l protocols. Fig. 6a shows the shelf lifetime of the PSC-Ref and PSC-2 stored in N_2_ atmosphere with 0% relative humidity (RH) at room temperature and at elevated temperature of 65 °C (Fig. 6b). Importantly, the PSC-2 showed a better stability compared to PSC-Ref (under ISOS-D1I), retaining more than 85% of initial PCE after 50 days of storage (PSC-Ref retained more than 85% of the initial PCE for only 20 days of storage) (Fig. 6a). The beneficial role of TaS_2_ protective buffer layer appeared also during ISOS-D2l stability tests (Fig. 6b), in which PSC-2 exhibited a superior thermal stability compared to PSC-Ref, retaining more than 60% of the initial PCE after 120 h (PSC-Ref retained only 43% of the initial PCE over the same time interval). The diffusion of the metal and of volatile species through the device and their reactivity to form metal halides have been recognized as the main intrinsic degradation pathway, which can be accelerated through heating.^77–80^ Thus, the improved thermal stability of the devices with TaS_2_ flakes can be directly correlated to a reduced diffusion rate of the volatile organic species, and a limited metal diffusion, both enabled by the TaS_2_ buffer layer.

To better understand the protection role of TaS_2_ layer, ISOS-D1 measurements were carry out by storing unencapsulated cells at ambient conditions at room temperature (Fig. 6c). Both PSC-Ref and PSC-2 showed higher degradation rate compared to those measured for storage at inert conditions (both devices retained only 20% of their initial PCE after 120 days of storage). It is evident that both devices showed similar degradation rate, losing 80% of their initial PCE after 100 days. This indicates that TaS_2_ flakes do not significantly protect the devices from ambient exposure (*i.e.*, humidity and oxygen) and thus, the protection mechanism observed in ISOS-L2, ISOS-D1I and D2I measurements must be of different nature. It should be noted that the thermal degradation of the PSCs is significantly faster compared to the degradation under room temperature and ambient conditions.

Based on these three different stability experiments, we concluded that the elevated operation temperature is a major degradation factor that reduces device lifetime from 120 days down to 120 h timescale.^81–83^ Even though TaS_2_ flakes fail to protect the unencapsulated PSCs operating at ambient conditions (Fig. 6c), the TaS_2_ buffer layer significantly improve the thermal stability of the device (Fig. 6a and b) stored under inert conditions (both at room temperature and 65 °C). Prospectively, our optimized TaS_2_-based PSCs can be further protected from external degradation factors such as humidity/oxygen through proper packaging^75,84^ while TaS_2_ buffer layer can solve the intrinsic instability issues.

Other buffer layers, such as AZO,^85,86^ TPBi,^87^ metal(acac)_*x*_,^88,89^ Zr(Ac)_4_ (ref. 90) have been successfully implemented in inverted PSCs between the PCBM and Ag layers. However, most of these studies focused on the PCE enhancement, without deeply discussing their effect on the device lifetime stability following an ISOS protocol. Comparing the stability enhancement of our approach with the literature, the TaS_2_ buffer layer improves the thermal stability of the devices and the lifetime of the devices is similar or even better. In this context, we recently reported the incorporation of 2D Bi_2_Te_3_ flakes buffer layer on top of PC_70_BM (doped with similar flakes), extending the lifetime of the perovskite devices, while retaining the 80% of their initial PCE for about 1100 h.^8^ In the current work, our interfacial engineering process leads to a similar enhancement of the device lifetime stability (our devices retain 80% of their initial PCE for 330 h) compared to our previous two-fold engineering strategy. Therefore, our results indicates that the incorporation of a 2D-material-enabled buffer layer over the PC_70_BM represents a viable route to improve the lifetime and thermal stability in inverted PSCs.

## Conclusions

The incorporation of TaS_2_ flakes as a protective buffer layer was demonstrated to improve all main PV parameters of PSCs. The systematic increase of the *V*_oc_ and *J*_sc_ is attributed to the passivation of the PC_70_BM surface traps upon buffer layer formation. This process reduces the non-radiative recombination while enhancing the charge extraction rate, verified by both PL and the transient measurements. By optimizing the spin coating deposition of TaS_2_ flakes, the champion devices reached a PCE as high as 18.45%, representing +12% PCE improvement compared to reference cells. More importantly, TaS_2_ buffer layer represents an effective thermal diffusion barrier against volatile species and metal ions, mitigating the intrinsic degradation pathways occurring in PSCs at elevated operational temperature (as verified by ISOS-L2 and -D2I stability tests). Overall, this novel interface engineering approach based on TaS_2_ buffer layer leads to extended thermal stability and longer device lifetime under continuous 1 Sun illumination and elevated temperature.

## Conflicts of interest

There are no conflicts to declare.

## Supplementary Material

NA-003-D1NA00172H-s001
