# Supplementary material for: Inverted perovskite solar cells with enhanced lifetime and thermal stability enabled by a metallic tantalum disulfide buffer layer
Source: Nanoscale Adv. 2021 Apr 9;3(11):3124–35. doi: 10.1039/d1na00172h (PMC9419768; doi:10.1039/d1na00172h)
Supplement: NA-003-D1NA00172H-s001 [file NA-003-D1NA00172H-s001.pdf]

Electronic Supplementary Material (ESI) for Nanoscale Advances.
This journal is © The Royal Society of Chemistry 2021

Supplementary material

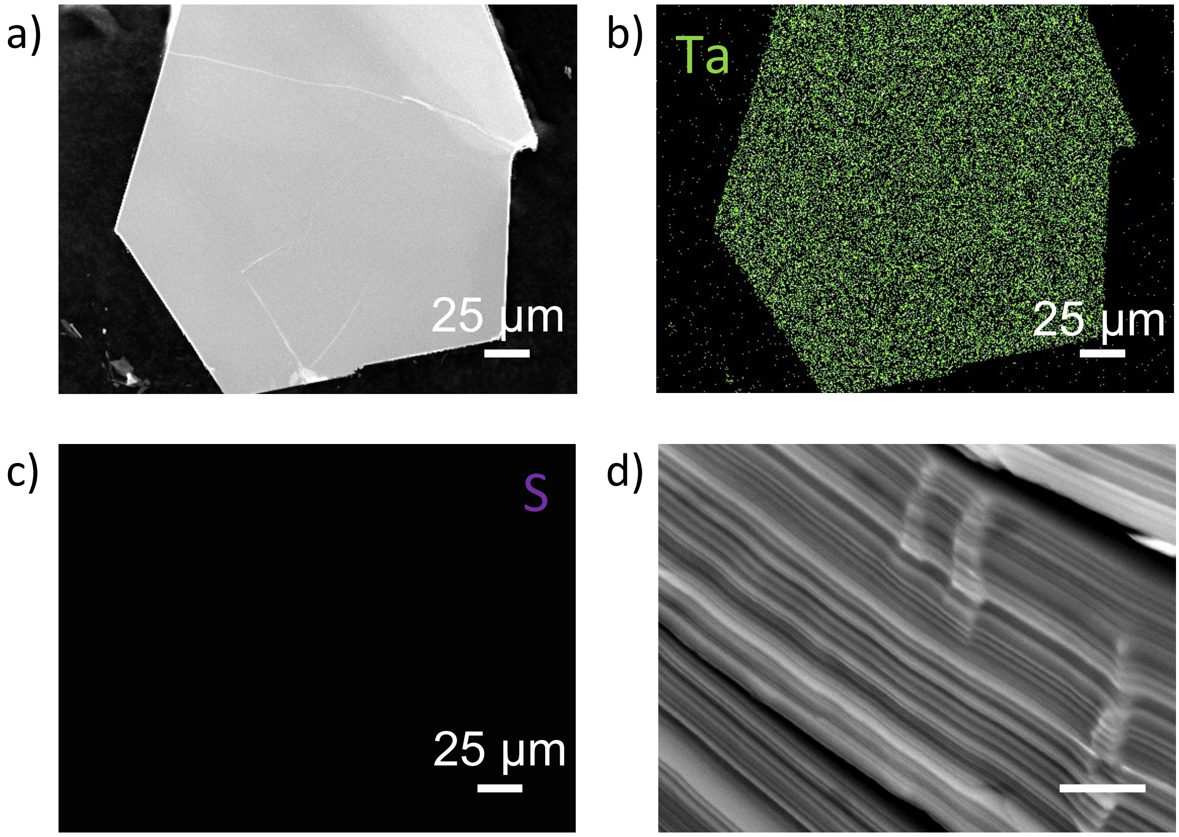

Figure S1. a) SEM image of the as-synthesized 6R-TaS_2 crystals and b,c) the corresponding EDS maps for Ta (M line at 1.71 keV, in green) and S (Kα line at 2.31 keV, in violet). d) High-magnification SEM image of an edge of a fragment of 6R-TaS_2 crystal, showing its layered structure.

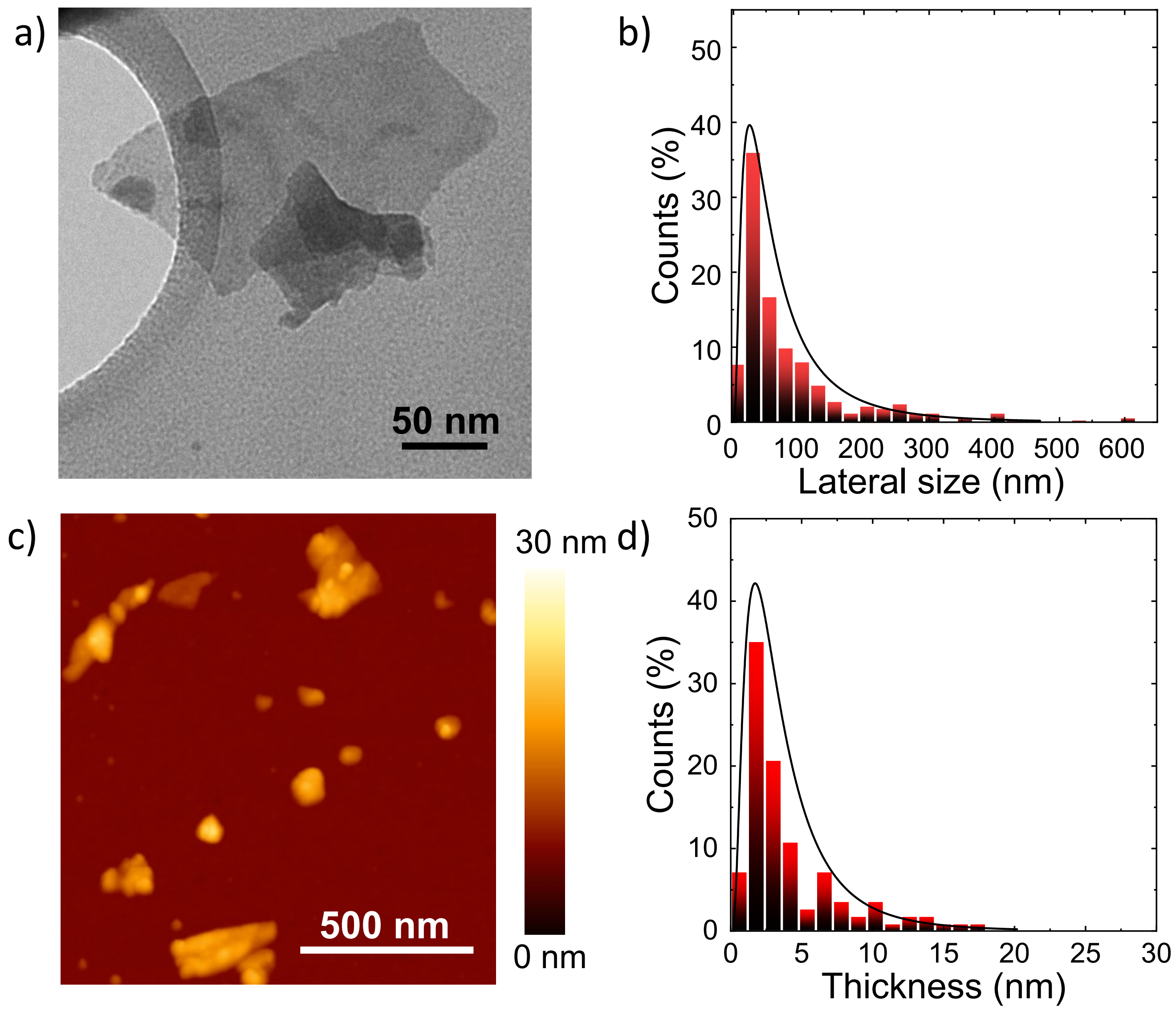

Figure S2. a) BF.TEM image of representative TaS_2 flakes and b) the statistical analysis of their lateral size (calculated on 300 flakes. c) AFM image of representative TaS_2 flakes and d) statistical analysis of their thickness (calculated on 110 flakes)

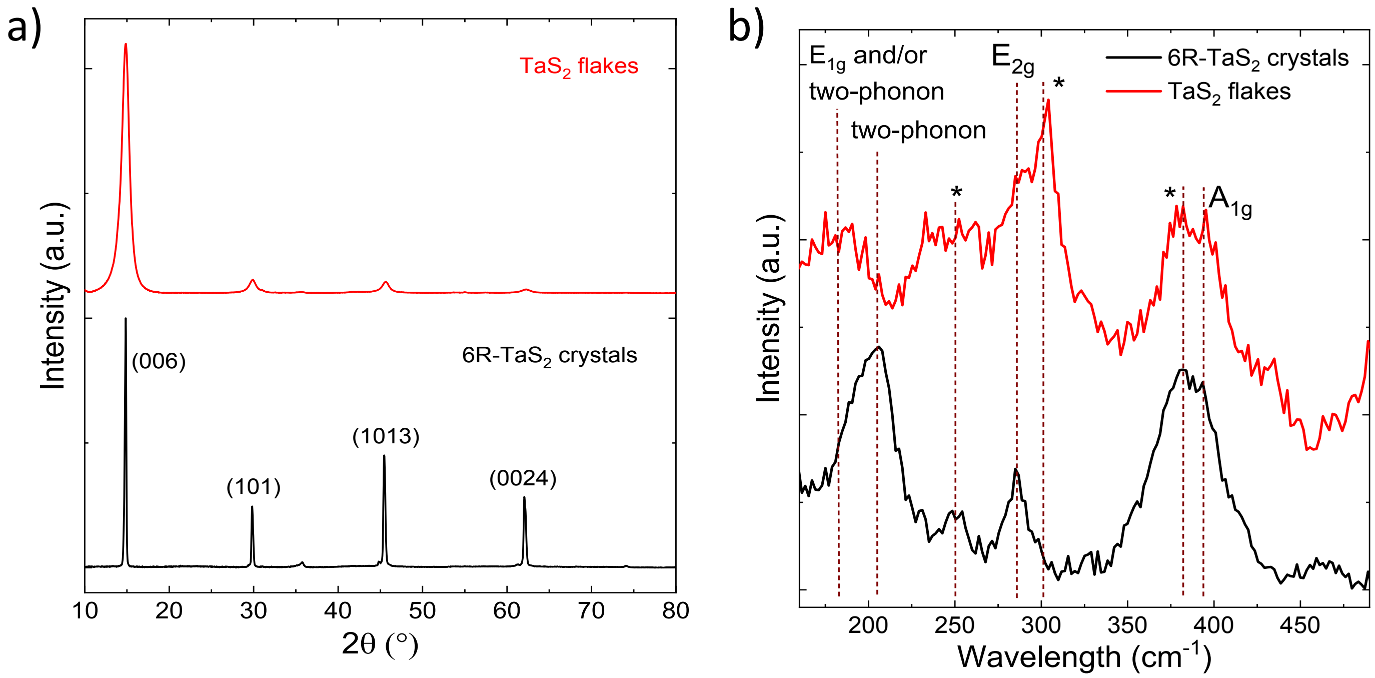

Figure S3. XRD spectra of the as-synthetized 6R-TaS_2 crystals and the TaS_2 flakes. b) Raman spectra of the as-synthetized 6R-TaS_2 crystals and the TaS_2 flakes. The Raman modes that have been identified for the 2H-TaS_2 are also denominated. The symbol * indicates possible fold-back optical modes attributed to the presence of layers with octahedral Ta coordination (similarly to those observed in 1T-TaS_2).

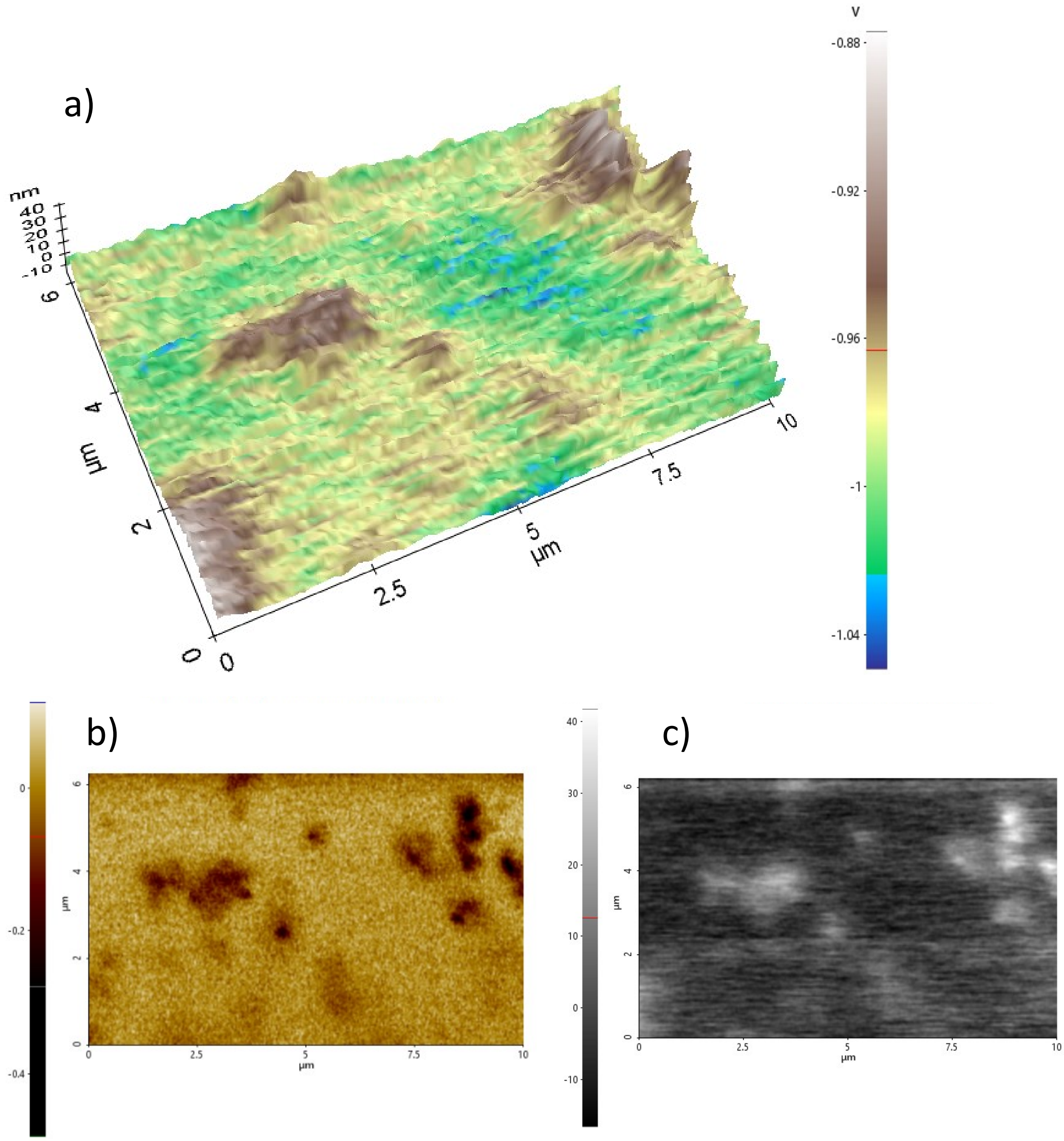

Figure S4. KPFM images of TaS_2 flakes spin coating on ITO. a) 3D representation of surface topography overlayed by KPFM contact potential, b) surface NC topography and c) NC phase image.

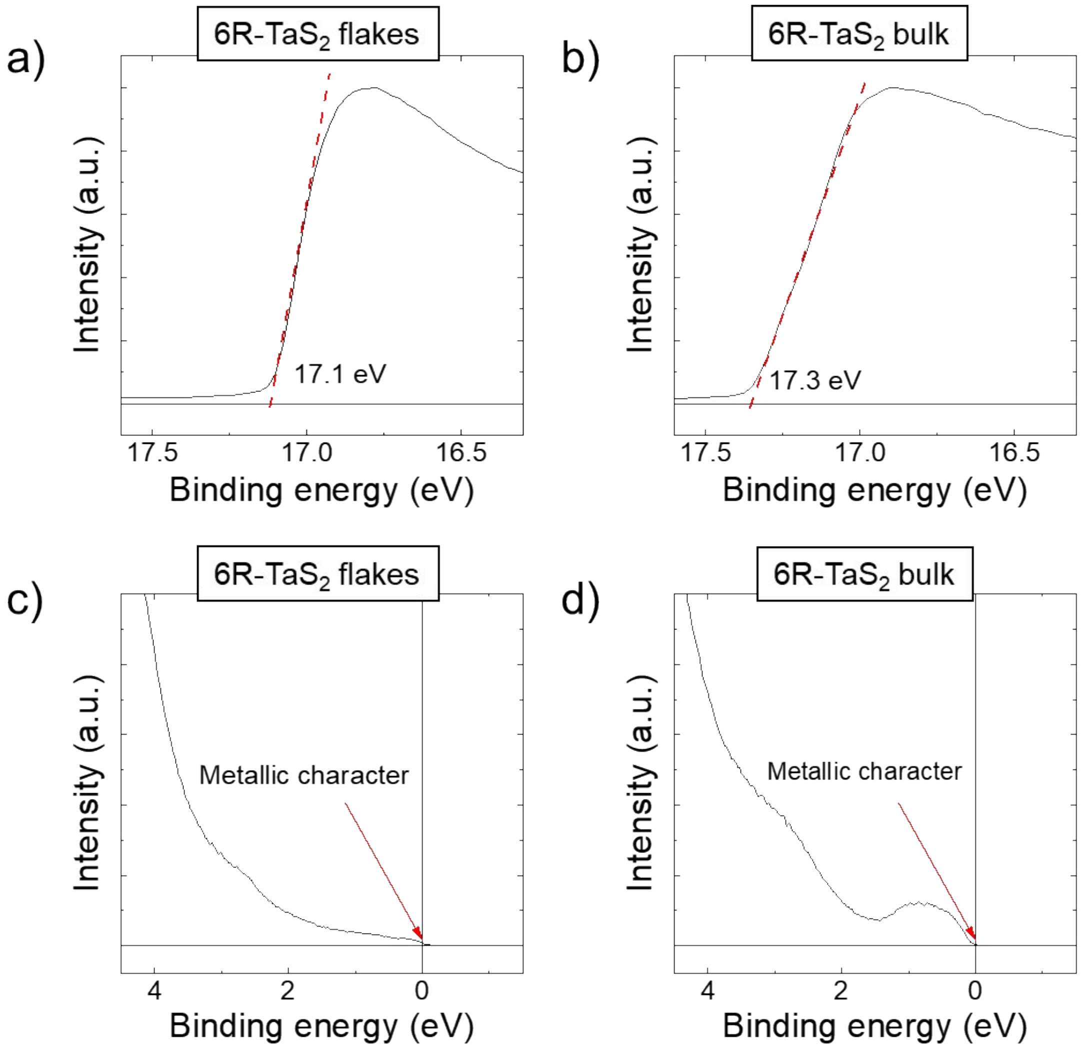

Figure S5. a,b) High binding energy regions of the ultraviolet photoelectron spectroscopy (UPS) spectra measured for 6R-TaS2 flakes and bulk crystals, respectively. c,d) Low binding energy region of the UPS spectra measured for 6R-TaS2 flakes and bulk crystals, respectively.

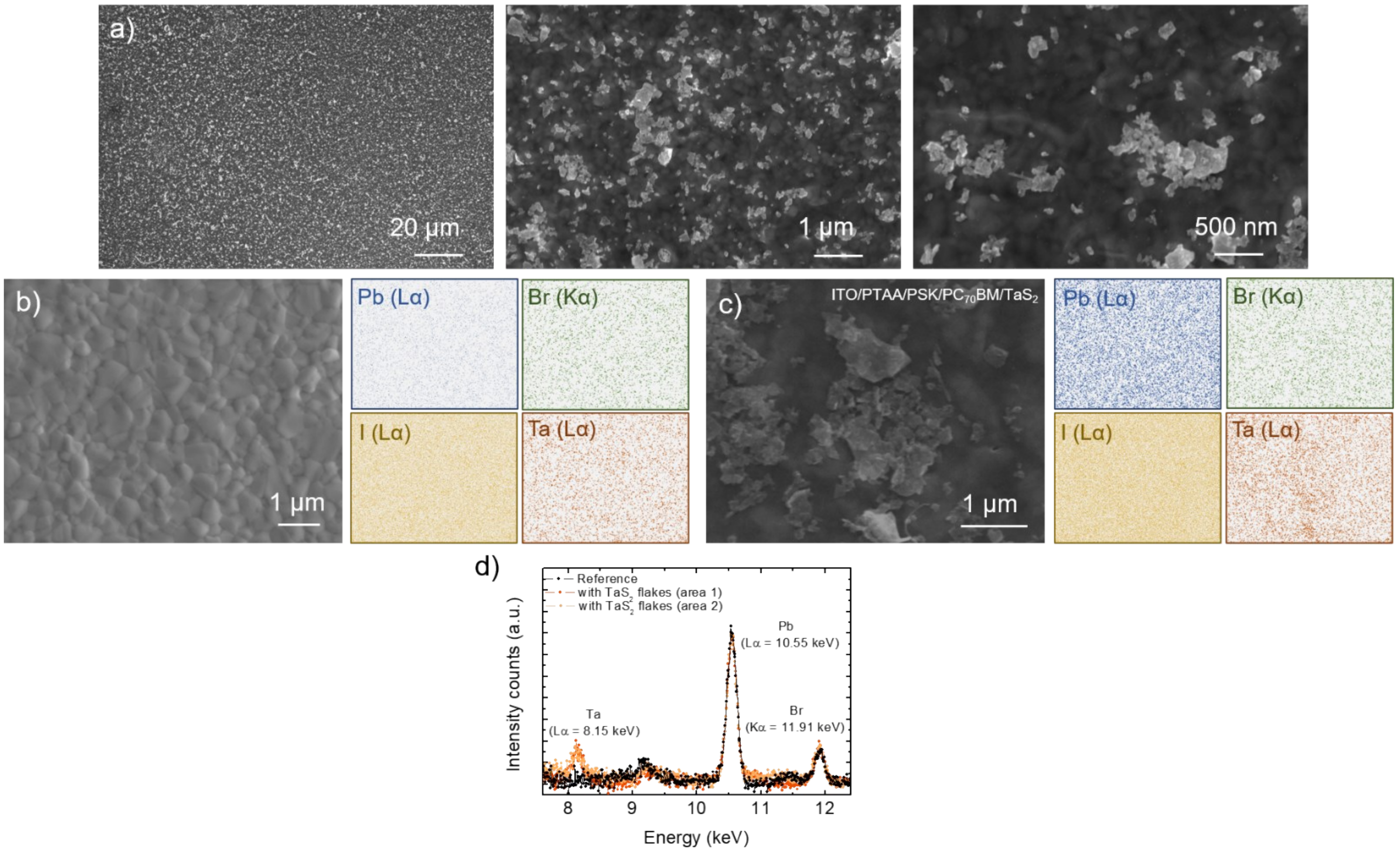

Figure S6. a) Low-magnification SEM images of the TaS_2 buffer layer deposited onto the $PC_{70}BM$ surface. b) SEM and EDS maps of the $PC_{70}BM$ and c) of the TaS_2 buffer layer deposited onto $PC_{70}BM$. d) Comparison of the EDS spectra collected for the $PC_{70}BM$ and TaS_2 enabled $PC_{70}BM$ at two different spots.

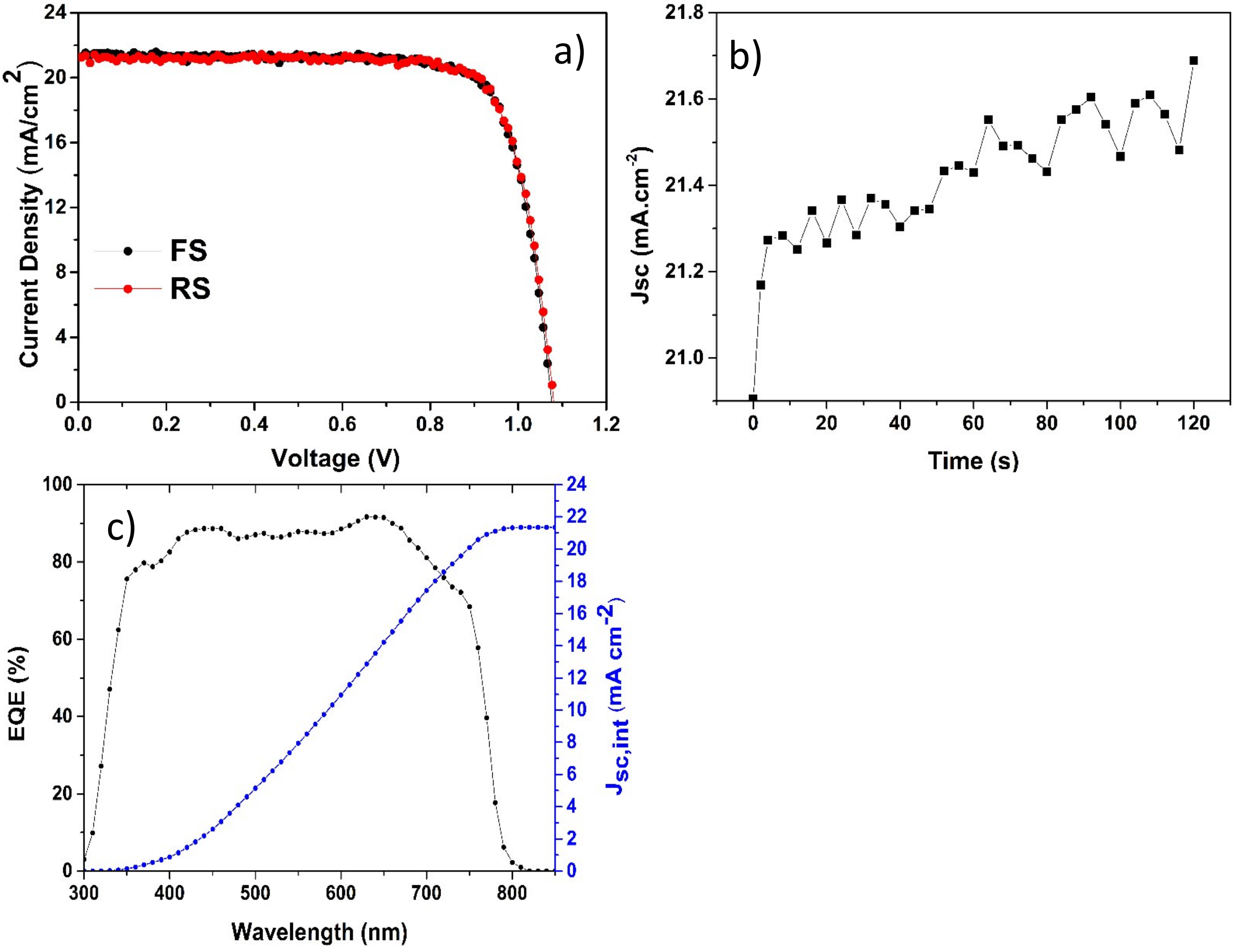

Figure S7. (a) J-V curves in forward and reverse scan directions of PSC-2 device revealing negligible hysteresis. (b) Evolution of photocurrent at sort circuit for 2 minutes. (c) EQE spectrum of a representative device and the integrated current density of the device's spectral response with the AM1.5G photon flux spectrum is depicted on the right axis.

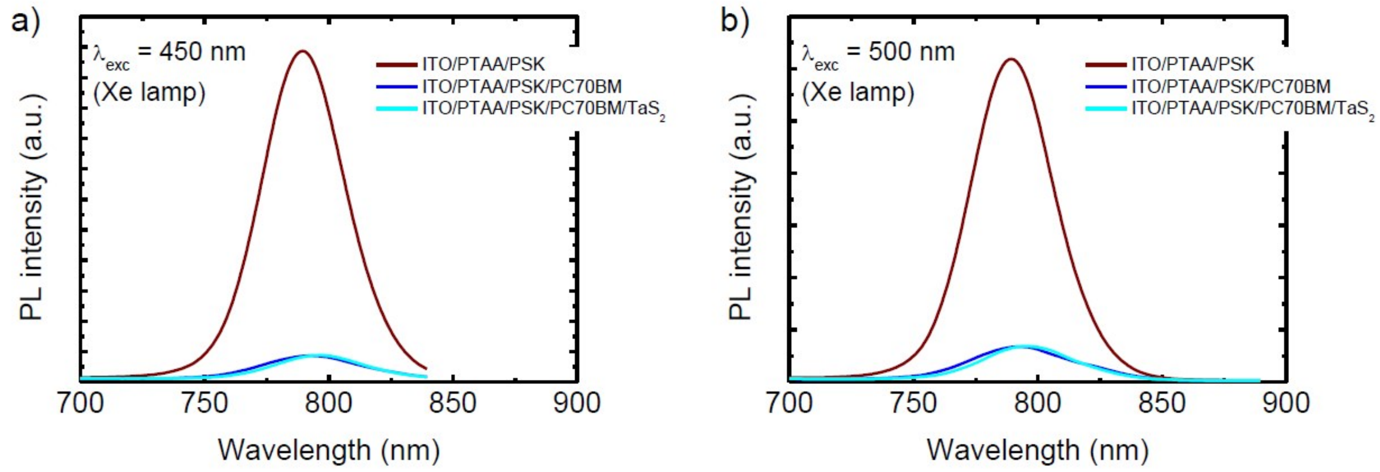

Figure S8. PL measurements of the structures ITO/PTAA/PSK, ITO/PTAA/PSK/$PC_{70}BM$, ITO/PTAA/PSK/$PC_{70}BM/TaS_2$ using (a) 450 nm and (b) 500 nm as excitation wavelengths (source Xe lamp in a LS-55 Perkin Elmer spectrofluorometer).

Table S1. Device PV characteristics extracted from the J-V curves in forward and reverse scan directions.

Sample	PCE (%)		V_{oc} (V)		J_{sc} (mA cm2)		FF	
	FS	RS	FS	RS	FS	RS	FS	RS
**PSC-Ref**	17.61	17.66	1.078	1.081	20.81	20.84	78.48	78.37
**PSC-1**	18.24	18.28	1.068	1.070	21.64	21.56	78.97	79.22
**PSC-2**	18.10	17.84	1.096	1.097	21.98	21.90	75.09	74.28
**PSC-3**	18.31	18.45	1.076	1.08	21.58	21.45	78.84	79.65

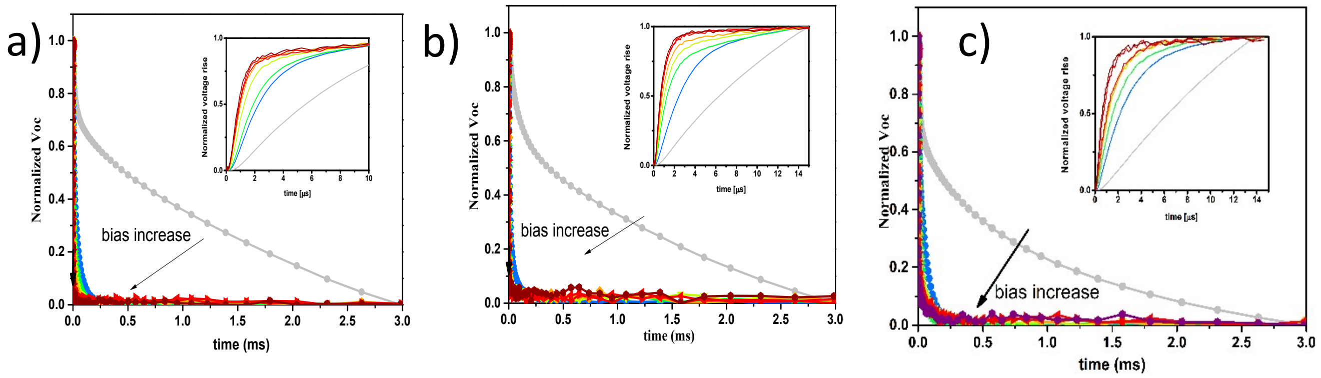

Figure S9. Raw data of TPV measurements for the investigated devices: a) Reference, b) PSC-1 and c) PSC-3. The insets present the voltage rise data.

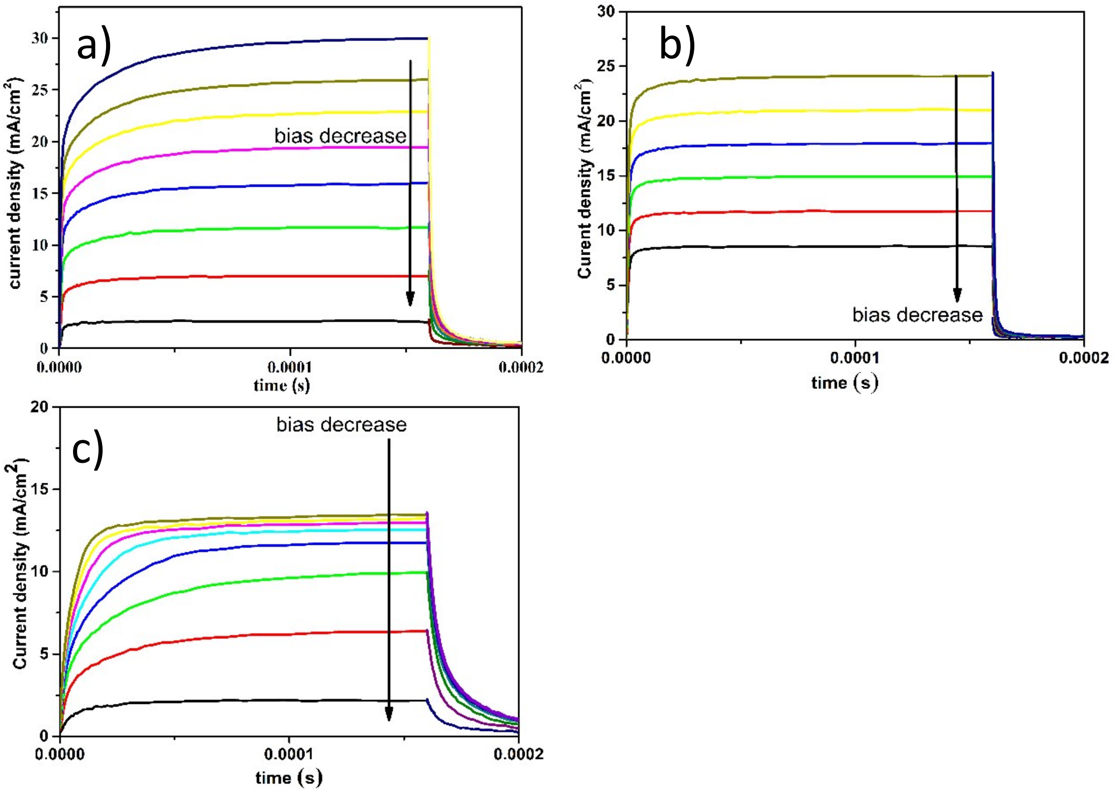

Figure S10. Raw data of TPC measurements for the investigated devices: a) Reference, b) PSC-1 and c) PSC-3.

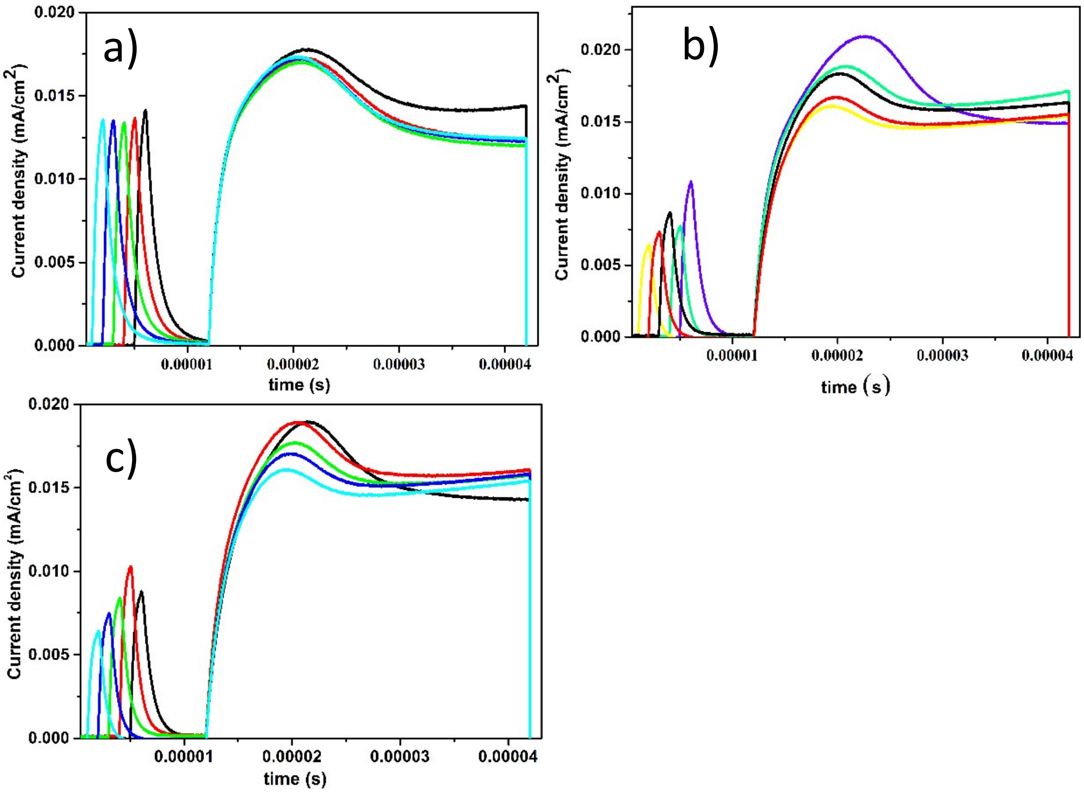

Figure S11. Raw data of photo-CELIV measurements used to extract the drift mobility for the investigated devices: a) Reference, b) PSC-1 and c) PSC-3.
